# Sources of variation for indoor nitrogen dioxide in rural residences of Ethiopia

**DOI:** 10.1186/1476-069X-8-51

**Published:** 2009-11-18

**Authors:** Abera Kumie, Anders Emmelin, Sonny Wahlberg, Yemane Berhane, Ahmed Ali, Eyassu Mekonen, Alemayehu Worku, Doris Brandstrom

**Affiliations:** 1 School of Public Health, College of Health Sciences, Addis Ababa University, Addis Ababa, Ethiopia; 2 Umeå International School of Public Health, Umeå University, Umeå, Sweden; 3 Addis Continental Institute of Public Health, Addis Ababa, Ethiopia; 4 Department of Pharmacology, Medical Faculty, Addis Ababa University, Addis Ababa, Ethiopia

## Abstract

**Background:**

Unprocessed biomass fuel is the primary source of indoor air pollution (IAP) in developing countries. The use of biomass fuel has been linked with acute respiratory infections. This study assesses sources of variations associated with the level of indoor nitrogen dioxide (NO_2_).

**Materials and methods:**

This study examines household factors affecting the level of indoor pollution by measuring NO_2_. Repeated measurements of NO_2 _were made using a passive diffusive sampler. A *Saltzman *colorimetric method using a spectrometer calibrated at 540 nm was employed to analyze the mass of NO_2 _on the collection filter that was then subjected to a mass transfer equation to calculate the level of NO_2 _for the 24 hours of sampling duration. Structured questionnaire was used to collect data on fuel use characteristics. Data entry and cleaning was done in EPI INFO version 6.04, while data was analyzed using SPSS version 15.0. Analysis of variance, multiple linear regression and linear mixed model were used to isolate determining factors contributing to the variation of NO_2 _concentration.

**Results:**

A total of 17,215 air samples were fully analyzed during the study period. Wood and crop were principal source of household energy. Biomass fuel characteristics were strongly related to indoor NO_2 _concentration in one-way analysis of variance. There was variation in repeated measurements of indoor NO_2 _over time. In a linear mixed model regression analysis, highland setting, wet season, cooking, use of fire events at least twice a day, frequency of cooked food items, and interaction between ecology and season were predictors of indoor NO_2 _concentration. The volume of the housing unit and the presence of kitchen showed little relevance in the level of NO_2 _concentration.

**Conclusion:**

Agro-ecology, season, purpose of fire events, frequency of fire activities, frequency of cooking and physical conditions of housing are predictors of NO_2 _concentration. Improved kitchen conditions and ventilation are highly recommended.

## Background

Biomass fuel is the primary source of household energy in developing countries. Fifty two percent of the global population and more than 90% of rural homes in developing countries use solid biomass fuels for cooking, heating, and lighting purposes [1]. Biomass fuel, also designated as unprocessed or dirty solid biofuel, mainly includes firewood, animal dung, agricultural residues and plant leaves.

Indoor air pollution is considered as one of the risk factors causing high burden of diseases and of premature deaths in developing countries [2-5]. IAP is recognized as a silent and unprotected killer among rural women and children who spend much of their time in the kitchen [6]. The burden of disease due to IAP is high in developing nations that use biomass fuels. Smith estimated that IAP in India accounted for 4.2-6.1% of the total national burden of disease, which is considered as a major public health concern. This proportion is tantamount to about half million premature deaths annually [7].

The health risk of direct exposure to biomass combustion is evident in part due to cooking practices in poorly ventilated and crowded single roomed houses in developing countries [4,8,9]. Studies have shown that proxy factors related to socio-economic status like income education, and use of biomass fuel to be highly associated with the level of IAP [7,4,10,11]. Despite the prevailing knowledge that exposure to biomass combustion products is high [4,9,12-19], quantitative studies on factors affecting the level of IAP are limited in developing countries [13].

Data on the measurement of indoor NO_2 _is limited in developed countries. Research has shown that the mean concentration of NO_2 _in kitchens with a gas stove varied between 26 parts per billion (ppb) and 112 ppb [20,21], while this was 18 ppb in kitchens equipped with electric stoves [20]. Median NO_2 _concentrations in living rooms were 5.8 ppb in Ashford (United Kingdom), 6.1 ppb in Minorca (Spain), 23.8 in Barcelona (Spain) [22] and 11 μg/m^3 ^(5.8 ppb) in Umea (Sweden) [23]. In low income homes of USA, mean concentrations of NO_2 _were found to be 43 ppb and 36 ppb in kitchens and living rooms, respectively [24] and 30 ppb in children's bedroom [25]. Increased level of NO_2 _was related with type of fuel and ventilation efficiency [26]. In addition, these studies consistently demonstrated that the type of fuel, purpose of fuel use, and location of indoor activities are associated with increased NO_2_. The use of gas stoves and heaters were often accompanied with exceeding the current World Health organization (WHO) Air Quality Guideline (AQG) of 21 ppb for 8 hours [27].

A particulate matter (PM) of various aerodynamic sizes is also used to measure the level of IAP. The mean concentration of respirable suspended particles (RSP) in homes that use biomass fuels had variation depending on the type of kitchen and fuel used: 500-2000 μg/m^3 ^in kitchens of India [28], 1200 μg/m^3 ^in Mozambique [29], 850-1560 μg/m^3 ^in Guatemala [16], and 1400 μg/m^3 ^in Kenya [30]. In Bangladesh, the average concentration of particulate matter (PM) of aerodynamic diameter of 10 microns (PM_10_) in kitchens that use biomass fuels ranged between 237 and 291 μg/m^3 ^[10]. Smith showed that the concentration of PM_10 _commonly varied between 200-5000 μg/m^3 ^[4]. In Ghana and Nicaragua, the average concentrations of PM_2.5 _varied between 320-650 g/m^3 ^in Ghana and Nicaragua [31,32]. These studies indicated that the type of kitchen (whether inside or outside), the type of stove (traditional or improved), ventilation condition, place of measurement (kitchen or sleeping room), and type of biomass fuel (wood, dung, or residues) were major factors affecting the concentrations of indoor air pollutants. The above cited aerial concentrations related to RSP, PM_10 _and PM_2.5 _exceeded by a factor of 2 to 40 times of the standards set by US Environmental Protection Agency, (US-EPA) for 24-hr and annual standards [33] and by a factor of 10 to 80 times of the present WHO 8 hr of air quality guideline (AQG) [27].

Biomass fuel in the form of firewood, agricultural residues and animal dung is the primary source of household energy in Ethiopia [34,35]. The majority of rural homes have only 1 room serving all types of household activities [36]. Cooking takes place in the same room using traditional unvented stoves. Such rooms do not have functional ventilation outlets [3,37]. Pocket studies in Ethiopia have shown high level of exposure to indoor air pollution that exceeded the WHO 1-hour and 8-hours AQG [27,38-40]. However, the results of these studies could not be generalized to a larger population due to their methodological limitations.

A recent study conducted by the authors revealed increased indoor NO_2 _concentration levels [41]. It also has shown that ecology and season were factors that affect NO_2 _concentrations. The present study was undertaken with an effort to further explore other factors associated with indoor air pollution in the rural Ethiopia. Given the high level of exposure to IAP in poorly ventilated housing units, increased morbidity and mortality due to acute respiratory infection (ARI) in the general population, especially among children under-five years old, are expected [3,42]. Therefore, this study will have great operational relevance in achieving the Millennium Development Goal 7 (MDG 7, target 9, indicators 27 & 29) by generating important information on feasible interventions for the reduction of IAP in the Ethiopian rural homes.

## Materials and methods

### Study setting, indoor air sampling and analysis for nitrogen dioxide

A longitudinal study was conducted to assess the level of indoor air pollution, by measuring NO_2 _level, in rural households over a period of 2 years (March 2000-April 2002) in a rural district (*Meskan and Mareko) *in mid-southern Ethiopia. The presence of a Demographic Surveillance System (DSS) that was instituted in the District since 1986 was an opportunity for the assessment of indoor NO_2 _concentrations. Indoor air samples for nitrogen dioxide were taken in approximately 3,300 homes with under five children.

NO_2 _was detected using a modified colorimetric *Saltzman *method. A 24 hours indoor air sampling was done by trained local enumerators at about 3 months interval to collect data on the date, start and finishing time (hh:mm) of each sample. A digital watch was used to record the time. NO_2 _concentration was measured using *Willems Badge *that was developed at the University of *Wageningen *in the Netherlands [43-45]. The polyethylene passive sampler consists of a small cylindrical cup equipped with 2 rings, chemically impregnated fibreglass placed at the bottom of the cup and Teflon to serve as a wind barrier. The sampler was set in a central wooden post of the rural housing (locally called "*tukul*") after ensuring the room was used for sleeping. The samplers in a batch were sent to the field after proper assembly. Sampling in the house was started by opening up the lid of the diffusive sampler (*Willems Badge*) and finished by closing it back after 24 hours of sampling.

Samplers from the field sites were transported to Addis Ababa where a centrally located laboratory was used for analysis. The absorption filter from the sampler was extracted with an acid solution of sulphanilamide and N-(1-naphtyl) ethylenediammonium dichloride (NEDA) which converts the absorbed nitrogen dioxide into NO_2_^- ^ions. The presence of these ions develops a red coloured solution whose absorbance was measured at 540 nm (Beckman DU^R ^.64 Spectrophotometer). A standard curve NO_2_solution was developed to calculate the NO_2 _concentrations using the following equation:

where:

'**C**' is indoor air concentration of NO_2 _in micrograms per cubic meter, '**mass**' is mass of NO_2 _on the glass fibre used for air sampling after subtracting mass of NO_2 _of the blank glass fibre, '**expmin**' is indoor air sampling duration in minutes, and '**40' **is a constant of air sampling rate for the diffusive sampler, 40 mlmn-1 [45].

The laboratory data quality was maintained using various methods. Laboratory technicians were properly trained and supervised on each day of laboratory analysis. Laboratory protocols were structured and monitored by standard practices. Internal validity were checked by the analysis of standard solution, blank absorbance, and control chart for NO_2_. The inter-laboratory variation was controlled by comparing the variations of NO_2 _concentrations of duplicate samples and exposed samples that were taken at different conditions.

Detailed description of the study area, sampling procedures, air sample location, and the analytical method is available elsewhere [41].

### Assessment of determinants of indoor nitrogen dioxide

A structured questionnaire was administered at household level immediately after the completion of the air sampling to collect fuel use related data and events that occurred at the time of NO_2 _sampling. Type of household fuel, purpose of having fire, type of cooked food and its timing were main variables collected during data collection. Ecological and seasonal factors were also considered due to their importance to affect indoor NO_2 _[41]. The physical dimensions (radius, axis, and wall height), the presence of window and separate kitchen in the study homes were extracted from the 1999 census data of the study setting.

### Data management and analysis

Data were entered and cleaned using the EPI INFO (version 6.04; Center for Diseases Control and Prevention, Atlanta, GA, USA and World Health Organization, Geneva, Switzerland). Consistency and completeness of each questionnaire was checked during data collection, entry and analysis. The data set was then exported first to data base file (DBF) and then to Statistical Package for Social Sciences (SPSS) (version 15.0; SPSS Inc., IL, USA) for advanced statistical analysis.

After data exploration, the original data set of indoor air NO_2 _concentration was transformed into logarithmic base 10 (log10) to meet the assumptions of normality for the analysis of variance (ANOVA) and linear mixed model regression analysis. In addition, box plots and stem plots were extensively used to observe the existence of outliers when comparing mean values of NO_2 _by categorical variables. Variables describing firing events were categorized in such a way to avoid multiple responses. These variables were type of fuel, purpose and time of fire events, and type of cooked food. One way ANOVA was employed for the detection of any differences and changes in the dependent variable represented by average indoor NO_2_concentration in the presence of categorical biomass fuel variables.

A mixed linear model was used to find out the relative importance of household characteristics on NO_2 _after ensuring assumption of normality in the dependent variable, linearity between dependent and independent variables, and collinearity between variables. A unique identifier for each household and the time variable attached to each NO_2 _measurement were created for this analysis. Ecology, season, type of biomass fuel, purpose and the time of the fire vents, and the frequency of food items were used for a fixed effect, while the quarterly measurements of NO_2 _were considered for the repeated effect. The intercept model was only used for the random effect as all households with under-five children were involved in the study. The use of unstructured covariance structure was found to be the best fit for the linear mixed model.

One way repeated measures of ANOVA was employed to analyze the presence of any difference in NO_2 _across time periods after structuring the data lay out into a long data format. The effect of housing characteristics (calculated indoor volume of each home, presence of window and kitchen) on indoor NO_2 _level was assessed using hierarchical model for a multiple linear regression analysis. Descriptive statistics, tables and inferential statistics were mainly used to present the findings. Further detailed management and data quality control are presented elsewhere [41].

## Results

### Characteristics of fuel use

**Figure 1:**
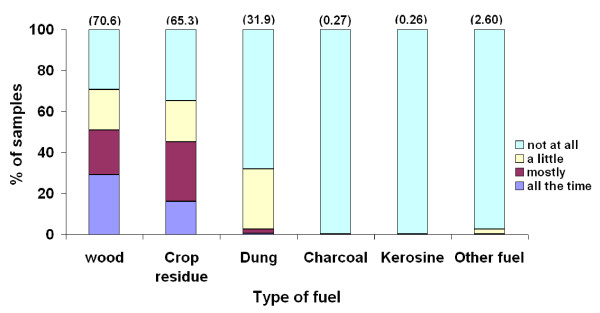
**Type of fuel and its use pattern that was observed during 24 hours of indoor NO_2 _sampling, Butajira, Ethiopia, 2000-2002 (n = 16899)**. For each type of fuel, the number in brackets indicates the proportion of type fuel used singly and in combination with others in reference to respondent's judgment comparing with the usual days.

The study was conducted for 2 years period involving 17215 indoor air samples in 3300 households with no refusal of participation. About 98% of air samples were taken at times of fire events in households. Biomass fuel in a form of wood, crop residues, and cow dung were largely used in 71%, 65%, and 32% of samples, respectively. In 2.6% of households, other type of fuels that were seasonally used including eucalyptus dry leaves, corncobs, and leaves of false banana. The use of mixed type of fuel was a common pattern (Figure 1). All firing events, whether for cooking or not, took place mainly indoors.

Cooking, lighting, heating, and insect repellent were reported as main reasons for having fire events in households in the last 24 hours during the time of air sampling (Table 1). Cooking foods and heating the space, (in 98% and 34% of the samples, respectively), were the major activities for fuel use. The use of biomass smoke for insect repellent was observed in approximately 13% of samples. Cooking and heating activities simultaneously took place in one third of the samples, while other activities in combination were rarely practiced representing less than 2% of the samples. Households had the practice of fire use three times a day. There were fewer activities at night that required the use of biomass fuel. Respondents in 73% of the samples perceived that firing at home took place relatively longer in the evenings than other times.

**Table 1:** Distribution of purpose of having fire and its timing that were observed during the 24 hours indoor NO_2 _sampling, Butajira, Ethiopia, 2000-2002 (n = 16899)

Purpose of Having fire	Morning # (%)*	Mid-day # (%)*	Evening # (%)*	Night # (%)*	Total # (%)*
Cooking	15670 (98.9)	14583 (99.1)	16325 (98.8)	934(94.9)	16622 (98.4)
Lighting	668 (4.2)	615 (4.2)	684 (4.1)	95 (9.7)	703 (4.2)
Heating	5529 (34.9)	5185 (35.2)	5718 (34.6)	331 (33.6)	5838 (34.5)
Insect repellent	1945 (12.3)	1527 (10.4)	2009 (12.2)	212 (21.5)	2122 (12.6)

**Total use of fire**	**15836 (93.7)**	**14714(87.1)**	**16520 (97.8)**	**984 (5.8)**	**16899**

*Percentages did not add up 100% due to multiple responses

With regard to cooked food items, kale cooking, traditional coffee ceremony, bread and local staple diet (locally called "*kocho*") baking were the usual type of traditional foods that were prepared during the 24 hours of indoor NO_2 _sampling. Traditional flat bread (locally called "*injera*") and its accompanying sausage (locally called "*wa*t") were rarely cooked, and only observed in less than 10% of the samples. Over 90% of cooking activities took place in the mornings and evenings, and rarely nights. Commonly cooked food items were kale ("*gomen*"), traditional coffee ceremony, and bread in 82%, 81% and 68% of samples, respectively. Other cooking activities were reported in 7.5% of the samples. These include roasting and boiling of peas and beans (locally called "kolo" and "*nifro*"), boiling of milk, cooking of cucumber and boiling of maize.

The association between fuel characteristics and ecological setting is presented in Table 2. Overall, there was a difference in the type of biomass fuel and its purpose of use. The use of wood predominated in the highland, while crop residues prevailed in lowlands. Heating of the housing space was more frequent in highlands (40%) than lowlands (28%). Cooking of any three food items and having three fire events per indoor air sampling day were commonly practiced in 73% and 80% of samples, respectively. Ecology was strongly related to all fuel use characteristics (p < 0.05).

**Table 2:** The characteristics of fuel use by ecological setting, Butajira, Ethiopia, 2000-2002 (n = 16899)

	Ecology type			
Characteristics	Highland, # (%)*	Lowland, # (%)*	P value for X^2^	Total # (%)*
**Type of fuel**				
Only wood	6209 (73.5)	2183 (8.5)	p < 0.0001	8392 (52.1)
Only Crop residue	2180 (25.8)	5231 (68.4)		7411 (46.0)
Only dung	63 (0.7)	234 (3.1)		297 (1.8)
**Total**	**8452 (52.5)**	**7648 (47.5)**		**16100**
**Purpose of having fire**				
Cooking	8905 (98.6)	7717 (98.1)	p < 0.0001	16622 (98.4)
Lighting	304 (3.4)	399 (5.1)		703 (4.2)
Heating	3609 (40.0)	2229 (28.3)		5838 (34.5)
Insect repellent	360 (4.0)	1762 (22.4)		2122 (12.6)
**Total**	**9031 (53.4)**	**7868 (46.7)**		**16899**
**Food frequency**				
Any one food item	318 (3.6)	331 (4.5)	p < 0.0001	649 (4.0)
Any two food items	1022 (11.6)	1423 919.2)		2445 (15.1)
Any three food items	6393 (72.9)	5433 (73.3)		11826 (73.1)
Any 4 & + food items	1040 (11.9)	224 (3.0)		1264 (7.8)
**Total**	**8773 (54.2)**	**7411 (45.8)**		**16184**
**Frequency of having fire**				
Any one time	147 (1.6)	175 (2.2)	p < 0.0001	322 (1.9)
Any two times	1088 (12.0)	1333 (16.9)		2421 (14.3)
Any three times	7386 (81.8)	6164 (78.3)		13550 (80.2)
Any four times	410 (4.5)	196 (2.5)		606 (3.6)
**Total**	**9031 (53.4)**	**7868 (46.6)**		**16899**

*Percentages did not add up 100% due to multiple responses

### Consistency of fire use events

There was not any difference in the time and frequency of fire use, type of fuel and type of cooked food items that occurred between the sampling time and 1 week recall period prior to that. It was only possible to identify that a religious holiday related to "*Romodan*" (the Moslem fasting month) was implicated to be a factor for additional cooking food items such as vegetable and meat soup, which took place relatively longer than the usual days of cooking.

### The level of NO_2 _by the characteristics of fuel use

The relative difference in NO_2 _concentration by proxy fuel factors is indicated in Table 3. The concentration of NO_2 _was found to significantly differ by type of fuel. On the average, households that used wood had geometric mean (GM) and geometric standard deviation (GSD) of 71.2 (2.8) μg/m3. These values for cow dung and crop residues were 67.5 (2.9) μg/m3 and 56.1 (2.7) μg/m3, respectively. Any combination of biomass fuel use did not significantly affect NO_2 _concentration.

**Table 3:** 24 hr indoor NO_2 _concentrations related to household characteristics in a bivariate analysis, Butajira, Ethiopia, 2000-2002 (n = 16899)

Characteristics	n	log NO_2_ mean(SD), μg/m^3^	GM (GSD), μg/m^3^	p-value	X^2 ^linear trend*
**Ecology**					
Highland	9018	1.89 (0.45)	77.2 (2.82)	p < 0.0001	
Lowland	7857	1.70 (0.43)	50.5 (2.67)		
**Total**	**16875**	**1.80 (0.45)**	**63.3 (2.81)**		
**Biomass fuel type**					
Only wood	8379	1.85 (0.45)	71.2 (2.8)	p < 0.0001	p < 0.0001
Only cow dung	297	1.83 (0.47)	67.5 (2.9)		
Only Crop residues	7400	1.75 (0.44)	56.1 (2.7)		
**Total**	**16076**	**1.80(0.45)**	**63.7 (2.8)**		
**Purpose of having fire**					
Any one activity	9256	1.84 (0.44)	69.2 (2.7)	p < 0.0001	p < 0.0001
Any two activity	6657	1.76 (0.46)	57.1 (2.9)		
Any three and above	886	1.75 (0.45)	55.6(2.8)		
**Total**	**16799**	**1.80 (0.45)**	**63.4 (2.8)**		
**Type of foods cooked**					
Any one food item	649	1.76 (0.46)	58.1 (2.9)	p < 0.0001	p < 0.0001
Any two food item	2442	1.74 (0.44)	54.7 (2.7)		
Any three food item	11806	1.81 (0.45)	65.3 (2.8)		
Any 4 food & above	1263	1.90 (0.43)	80.0 (2.7)		
**Total**	**16160**	**1.81 (0.45)**	**64.3 (2.8)**		
**Time of having fire**					
Any one time	322	1.50 (0.52)	30.8 (3.3)	p < 0.0001	p < 0.0001
Any two time	2418	1.75 (0.42)	56.2 (2.6)		
Any three time	13530	1.82 (0.45)	65.7 (2.8)		
Any four times	605	1.82 (0.42)	66.2 (2.6)		
**Total**	**16875**	**1.80 (0.45**)	**63.3 (2.8)**		
**Season**					
Dry season	8160	1.78 (0.45)	59.64 (2.8)	p < 0.0001	
Wet season	8715	1.83 (0.45)	70.0 (2.8)		
**Total**	**16875**	**1.80 (0.45**)	**63.3 (2.8)**		

*Linear trend was using EPI INFO version 6.04 STATCALC calculator Chi for linear trend. Those who used and not used the respective fuel type were considered as cases and controls.

The GM (GSD) concentration of NO_2 _representing a single purpose of having a fire in a household was 69.2 (2.7) μg/m3, any two purposes was 57.1 (2.9) μg/m3 and any three or more purposes was 55.6 (2.8) μg/m3. Multiple comparisons indicated that high level of NO_2 _was related to only single purpose compared to combination of them (p < 0.05). The concentration of NO_2 _had a declining linear trend from a single activity to combined activities (p-value for linear trend (p < 0.05).

Multiple food cooking was strongly related to NO_2 _indoor level compared to any single food preparation (p < 0.05). An increasing linear trend with the number of cooked food was also observed (p-value for linear trend (p < 0.05). Coffee drinking as well as bread and "Kocho" baking were the usual types of food that were frequently cooked.

Increased level of NO_2 _was significant among households that frequently used firing (p < 0.05). One time of fire use per day was related to GM (GSD) NO_2 _of 30.8 (3.34) μg/m3, while this was 64.3 (2.79) μg/m3 for any combination of timing of fire in reference to the morning, daytime, evening or nighttime. NO_2 _had an increasing trend with the frequency of cooking time (p-value for linear trend (p < 0.05).

In a mixed model linear regression, type of ecology, season, type of fuel, frequency of fire events and number of foods cooked per day were able to explain overall variations in NO_2 _concentrations (Table 4). A household being in a highland, wet season, use of crop residues, any time of having a fire event, frequency of food items, and interaction between ecology and season emerged as predictors of indoor NO_2 _concentration. The purpose of fire events did not make any effect.

**Table 4:** Estimates of fixed effects of fire use characteristics in households having fire events during last 24 hours, Butajira, Ethiopia, 2000-2002 (n = 3849)

					95% Confidence Interval of slope	
					
Indoor Fire use characteristics	Parameter Estimate (Slope)	Std. Error	T	p-value	Lower Bound	Upper Bound
**Intercept**	1.806	0.034	53.48	p < 0.0001	1.740	1.872
**Ecology**						
Highland	0.189	0.012	15.75	p < 0.0001	0.165	0.212
Lowland	**†**					
**Season**						
Dry	-0.030	0.009	-3.33	p < 0.001	-0.048	-0.012
Wet	**†**					
**Biomass fuel type**						
Only wood	-0.019	0.025	-0.79	0.432	-0.067	0.029
Only Crop residues	-0.048	0.024	-1.98	0.047	-0.096	-0.001
Only dung	**†**					
**Purpose of fire events**						
Cooking	0.026	0.016	1.65	0.098	-0.005	0.057
Heating	-0.029	0.016	-1.87	0.061	-0.060	0.001
Insect repellent	**†**					
**Frequency of fire events**						
One time per day	-0.146	0.032	-4.52	p < 0.0001	-0.209	-0.083
Two times per day	-0.00001	0.019	-0.001	0.999	-0.038	0.038
Three times per day	0.020	0.017	1.16	0.247	-0.014	0.054
Four times per day	**†**					
**Frequency of food items**						
One food item	-0.068	0.021	-3.32	p < 0.001	-0.109	-0.028
Two food items	-0.093	0.015	-6.39	p < 0.0001	-0.122	-0.065
Three food items	-0.050	0.012	-4.09	p < 0.0001	-0.074	-0.026
Four food items	**†**					
Highland * Dry season	-0.066	0.012	-5.39	p < 0.0001	-0.090	-0.042
Highland * wet season	**†**					

Dependent Variable: indoor concentration in linear scale transformed to log 10; **†: **Reference group

### Level of NO_2 _by time of measurement and housing structure

The time for NO_2 _measurement from our database and variables on housing (calculated volume, window, and kitchen) from Butajira DSS database were extracted for the analysis. The mean (SD) NO_2 _measurements during our study period was 4.37 (1.90) per household, while the median was 5. Nearly 70% and 56% of the households had at least 4 and 5 measurements of NO_2_, respectively. There was a significant difference in the repeated measurements of NO_2 _concentrations over time [Wilk's Lambda = 0.993, (F(4,2047) = 3.46, p < 0.05 with multivariate eta squared = 0.007 and observed power of 89%]. The five NO_2 _measurements used for comparison in ascending order of time 1 to time 5 (n = 2186) had GM (GSD) as follows: 68.6 (2.58), 70.3 (2.54), 69.1 (2.47), 70.1 (2.49), and 65.2 (2.62) μg/m^3 ^(n = 2051) Post hoc pair-wise comparison showed the overall difference was accounted between time 2 and time 5, and time 4 and time 5. The repeated NO_2 _measurements also differed by ecology, (F(1,2049) = 260.5, p < 0.05) and by location of households (locally called '*peasant associations'*), [F(8,2042) = 48.9, p < 0.05)].

The calculated volume of "*tukul*" was linearly related to NO_2 _concentration [β (95% CI): 0.104 (0.055, 0.153]. The addition of window in the 2^nd ^model did not show any association, while kitchen in the 3^rd ^model showed a significant relationship with indoor NO_2_. The indoor volume showed positive relationship, while kitchen was negatively related to indoor NO_2. _Indoor volume alone indicated NO_2 _to vary by about 1.0 μg/m^3 ^for every 10 m^3^. Both volume and kitchen were able to explain less than 1% variations in NO_2 _[adjusted R^2 ^= 0.008] (Table 5).

**Table 5:** A relationship between indoor NO_2 _and housing physical structures, Butajira, Ethiopia, 2000-2002 (n = 2882)

Model	Variable	Regression Coefficients (Beta), (95%CI)	Std. Error of Beta	t- statistics	p-value for beta	Model R^2^ (adjusted)	Standardized beta
**1**	Volume	0.104 (0.055,0.153)	0.025	4.16	p < 0.0001	0.006	0.077
**2**	Volume	0.112 ((0.061,0.162)	0.026	4.35	p < 0.0001	0.006	0.083
	Window	-0.018 (-0.046,0.010)	0.014	-1.28	0.202		-0.024
**3**	Volume	0.102 (0.51,0.152)	0.026	3.93	p < 0.0001	0.008	0.076
	Window	-0.022 (-0.05,0.005)	0.014	-1.58	0.115		-0.030
	Kitchen	-0.5.3 (-0.89,-0.018)	0.018	-2.94	p < 0.01		-0.055
**Condensed** **model**	Volume	0.093 ((0.043,0.142)	0.025	3.66	p < 0.0001	0.008	0.069
	Kitchen	-0.050(-0.085,-0.015)	0.018	-2.79	p < 0.01		-0.052

## Discussion

Selected household characteristics affecting the level of indoor air pollution have their own role in changing the level of NO_2 _in the context of our study area. Nearly all households in the study area used biomass fuel in the form of firewood, crop residues, and animal dung among which the first two predominated. The population in the study area is known for using wood most of the time throughout the year, crop residues such as stocks of maize and barley during harvest times, and animal dung during summer [46]. While biomass fuel sources are relatively cheap and easily available locally, fuels of fossil origin such as kerosene was only used to light local lamps for the interior of housing units at night. Kerosene is less affordable for rural residents to use it for cooking purposes compared to residents of urban areas such as Addis Ababa [47]. The use of biomass fuel as a primary source of household energy is consistent with findings of studies in other developing countries [1,10,19,28,38,48].

Biomass fuel was extensively used for cooking traditional foods compared to other purposes of having fire events. Cooking was the most frequent household fuel use activity reported during all times of the day. Heating was the second most frequent activity; whereas only about a tenth of the households used biomass fuel to repeal mosquitoes at night. The use of heating indoor space predominated in colder villages, mainly in the highlands, while repealing mosquitoes prevailed in the lowlands. It is evident from the data that home heating in the highlands might have caused an additional fuel use burden, which possibly contributed to the increased concentration of indoor NO_2 _compared to the relatively low fuel use burden required for repelling insects in the lowlands. The extreme temperature difference [49] might have contributed to the variation in the use of fuel for heating between the two ecological settings. Low temperature in early mornings and nights is common in the highlands of Ethiopia. Villages in the lowlands inherently possess a risk to malaria caused by mosquito bites and, therefore, there is a cultural practice in the study area for using indoor firing events to repel mosquitoes [42,50].

Traditional foods that do not require a stock for more than a day were routinely cooked. The cooking time was equally important in all cases of cooking which involved commonly the mornings, mid-days and evenings. Traditional coffee and bread making are also common daily practices in the study area. Coffee in each household was served for a group of neighborhoods nearly on daily basis, which is a cultural heritage of Ethiopia. The relative time and cost of preparing these food items are a bit less than that for "*injera*" and "*wat*" which are widely used in other parts of Ethiopia, especially in the temperate and highland areas. The practice of "*injera*" and "*wa*t" is expensive and the raw material, locally called "*teff*" is considered as a cash crop for the rural residents in the study area. The linear relationship between the number of food items cooked and frequency of cooking with the level of IAP is obvious given the increased respective amount of biomass fuels and the corresponding higher emission of other pollutants including nitrogen dioxide. This is in part a reflection of "dose-response" relationship.

Cooking and heating are the main household activities that lead to the excess NO_2 _concentration due to solid biomass fuel use in general, and in the highland areas in particular. In other studies, given the range of the purpose of biomass fuel use, cooking has been implicated as the main factor for the greater proportion of exposure to IAP in developing countries [4,51]. Biomass fuel emits about 50 times more pollution during cooking compared to cleaner fuels [4], while the exposure magnitude of breathing in pollutants could be twice more for the same population [7]. Therefore, the magnitude of health risk due to biomass combustion can reach as much as 2-3 times greater than the risk among clean fuel users [4,9]. Indoor smoke from biomass fuel is attributed to loss of healthy life in poor countries due to known health outcomes such as ARI, acute lower respiratory infections, and chronic obstructive lung diseases [52]. It is possible to speculate based on our findings that higher degree of exposure to indoor air smoke goes to mothers and children who often spend most of their time indoors. This implies that the attainment of Child Health MDGs would be a challenge in developing countries, like Ethiopia.

Assumption of the within-subjects (within a household over time) variation in indoor NO_2 _concentration by time was certainly important given the possible differences in the exposure to various fuel characteristics within households. This determined the presence of differences in exposure factors. The repeated between subject difference in NO_2 _demonstrated by ecological factors and seasonality is an important effect that requires closer attention for designing an appropriate intervention, as well. The study revealed only an interaction between ecology and seasonality affecting the indoor NO_2 _concentration by time. This was supported by our data that indicated variations of NO_2 _to be dependent on the type of fuel in the bivaraite analysis, although this association remained significant for a crop residue in a linear mixed model.

Ecology, season, type of biomass fuel, the purpose and time of having fire events, and the frequency of cooked food items were all found to affect the level of indoor air in a bivariate analysis, while this association was consistent in a linear mixed model except for the type of biomass fuel for wood. In western countries, the type of fuel (gas or electricity), occupancy density, the number of cooked meals, frequency of cooking, season and income were found to significantly impact indoor NO_2 _concentrations [20,24-26,53]. Those studies have indicated the presence of strong link among factors responsible for the increased level of indoor NO_2 _both in the developed and developing countries. The new finding in our study is the presence of ecology as a factor that predominately affected the level of NO_2_. The high level of NO_2 _in the highland areas can also be explained by high proportion of wood fuel use. Wood is at least better than crop residues and animal dung in the energy ladder [54] and provides relatively better energy efficiency. When wood is used, it oxidizes relatively more indoor air nitrogen because of the relatively high combustion temperature than others.

The effect of housing volume was found to show little importance in affecting IAP as measured by NO_2_, which was against our hypothesis. Together with the presence of window and kitchen in the multiple linear models, there was only very small proportion of explainable variance (less than 1%) in NO_2 _concentration despite the statistical significance. The computed model was not able to indicate a practical relevance in explaining the direction and strength of the association between the magnitudes of indoor air pollution and the physical housing characteristics in our study area. The significant difference, however, could only be explained due to large sample size that could have picked up small differences for calculating p-value. Rural housing units, due to their nature of construction, allow the easily passage of indoor smoke through their thatched roof, open eves and unplastered or partially plastered wall, restricting the continued built up of indoor air pollutants. It is quite common to observe visually the penetration of intense smoke through such structures during active cooking times in early mornings when there is good visual contrast (personal observation). Windows in the majority of housing units in the area are represented by just small circular holes (usually < 5% of the floor area, which is often closed due to the fear of wind drafts). Furthermore, opening of windows is culturally believed to affect resident's heath in our study settings.

Lack of assessment for additional air pollutants such as PM and the absence of real time measurement for the indoor air pollution were major limitations of this study. Nevertheless, the present study has shown that ecology, season, purpose of fire events, the frequency of cooked food items and the frequency of fire events as being predictors of indoor NO_2 _in a rural setting.

## Conclusion

Agro-ecology, season of the year, fire use characteristics and the physical structure of the housing were found to be important determinants of indoor air pollution in this study.

Further study on personal exposure assessment using NO_2 _and PM is highly recommended. In addition, relating indoor NO_2 _levels with commonly seen childhood diseases, such as respiratory symptoms, is another area of significant relevance for a research given the high level of IAP in our study settings. Finally, the provision of separate kitchen, improved stoves with hood, and presence of window in kitchens are highly advised to manage low level of IAP.

## List of abbreviations

AQG: ambient air quality; DSS: Demographic Surveillance System; EPA: Environmental Protection Agency; GM: geometric mean; GSD: geometric standard deviation; g/m^3^: gram per cubic meter; IAP: indoor air pollution; MDG: Millennium Development Goal; μg/m^3^: micro grams per cubic meter; mlmn^-^: millilitre per minute; NEDA: N-(1-naphtyl) ethylenediammonium; NO_2_: nitrogen dioxide; PM: particulate matter; PM_10_: aerodynamic diameter of 10 microns PM; ppb: parts per billion; ppm: parts per million; RSP: respirable suspended matter; WHO: World Health Organization.

## Competing interests

The authors declare that they have no competing interests.

## Authors' contributions

AK and AE were involved in the study protocol design development, data collection, data quality monitoring, data analysis and preparation of the manuscript. YB and AA were involved in analysis and editing draft manuscripts. SW and DB designed, trained, and supervised NO_2 _data collection and laboratory analysis. EM was involved in supervising and monitoring laboratory data analysis. AW was involved mainly in data analysis and its data quality management. All authors contributed to revising the final manuscript.
